# Silicon Nanoparticles Enhance Ginger Rhizomes Tolerance to Postharvest Deterioration and Resistance to *Fusarium solani*

**DOI:** 10.3389/fpls.2022.816143

**Published:** 2022-03-15

**Authors:** Huimin Peng, Haijun Hu, Keyong Xi, Xiongmeng Zhu, Jie Zhou, Junliang Yin, Fengling Guo, Yiqing Liu, Yongxing Zhu

**Affiliations:** ^1^Hubei Key Laboratory of Waterlogging Disaster and Agricultural Use of Wetland, College of Horticulture and Gardening, College of Agriculture, Yangtze University, Jingzhou, China; ^2^Institute of Economic Crops, Hubei Academy of Agricultural Sciences, Wuhan, China

**Keywords:** SiNP, Zingiber officinale Roscoe, antioxidant enzyme activities, water loss, MYB, AQP

## Abstract

Postharvest deterioration of ginger rhizome caused by microorganisms or wound infections causes significant economic losses. *Fusarium solani* is one of the important causal agents of prevalent ginger disease soft rot across the world. The massive and continuous use of chemical fungicides in postharvest preservation pose risks to human health and produce environmental contamination. Hence, new alternative tools are required to reduce postharvest deterioration and extend the postharvest life of ginger. In this study, the use of silicon nanoparticles (SiNPs) on the storability of ginger rhizomes during postharvest storage and their resistance to *Fusarium solani* was investigated. The results showed that 50, 100, and 150 mg L^−1^ of SiNPs increased the firmness of the ginger rhizome during storage but decreased the decay severity, water loss, total color difference, and the reactive oxygen species (ROS; H_2_O_2_ and superoxide anion) accumulation. Specifically, 100 mg L^−1^ (SiNP100) demonstrated the best effect in the extension of postharvest life and improved the quality of the ginger rhizomes. SiNP100 application increased the activities of antioxidant enzymes (SOD and CAT) and the total phenolics and flavonoid contents, thereby reducing the ROS accumulation and malondialdehyde (MDA) content. Meanwhile, SiNP100 treatment negatively impacts the peroxidase (POD) and polyphenol oxidase (PPO) activities, which may have contributed to the lower level of lignin and decreased total color difference. SiNP100 likely decreased water loss and the transfer of water by altering the expression of aquaporin genes. Moreover, SiNP100 modulated the expression of lignin synthesis and phytopathogenic responses genes including MYB and LysM genes. Furthermore, SiNP100 inhibited *Fusarium solani* by preventing the penetration of hyphae into cells, thus decreasing the severity of postharvest pathogenic decay. In summary, this study revealed the physiology and molecular mechanisms of SiNPs-induced tolerance to postharvest deterioration and resistance to disease, which provides a foundation for using SiNPs resources as a promising alternative tool to maintain ginger quality and control postharvest diseases.

## Introduction

Ginger (*Zingiber officinale* Roscoe) is one of the most economic important vegetables in the Zingiberaceae family, and it is cultivated in many tropical and subtropical countries such as China, India, and Australia (Liu et al., 2016). Its rhizome contains a large number of antioxidants and polyphenols, including flavonoids and flavones glycosides (Kaushal et al., 2017). Therefore, ginger has been widely used as a food, spice, flavoring agent, and medicine due to its beneficial characteristics such as aroma, pungency, nutrients, and medicinal properties. However, similar to most vegetables, ginger deteriorates rapidly after harvest due to dehydration, pathogen invasion, and senescence. This seriously decreases the quality, flavor, and medicinal effect of rhizomes during postharvest storage and results in significant economic losses (Kaushal et al., 2017; Li et al., 2020). Thus, there is a need to reduce decay and extend the postharvest life of ginger.

The most common strategy in postharvest preservation is the use of synthetic chemical fungicides, but the massive and continuous use of these chemicals leads to several problems, such as chemical residues that pose risks to human health and produce environmental contamination (Palou, 2018). Hence, the scientists are searching for new alternative tools to maintain postharvest quality (Ardakani, 2013). To date, various eco-friendly postharvest treatments including chitosan, oligochitosan (Liu et al., 2016), eugenol (Hu et al., 2016), humic acid (Sheteiwy et al., 2017), and methyl jasmonate (Nawaz et al., 2017; Li et al., 2019) have been applied for the postharvest preservation of vegetable and fruit as well as for stress alleviation. Si (silicon) is generally regarded as a safe substance by the United States Food and Drug Administration and has been used as abiotic elicitor to control postharvest diseases (Romanazzi et al., 2016). Exogenous sodium metasilicate and nano-Si treatment have been tested as effective postharvest treatment to reduce postharvest disease and delay postharvest decay in several species including sweet cherry (Qin and Tian, 2005), potato tubers (Li et al., 2009), Chinese cantaloupe (Liu et al., 2009), citrus fruits (Liu et al., 2010), and carrot (Elsherbiny and Taher, 2018). Sodium silicate has been reported to prime defense responses by regulating mitochondrial energy metabolism and reactive oxygen species (ROS) production in pathogen infected muskmelon (Lyu et al., 2019). Elsherbiny and Taher (2018) proposed that silicon could decreased the incidence and severity of carrot sclerotinia rot during storage. Based on the aforementioned research, silicon-induced plant resistance could be attributed to the following mechanisms (1) the formation of physical or mechanical barriers preventing water loss and fungal penetration; (2) elicitation of defense-related responses at both the enzyme-activity and transcript levels and induction of biochemical defense reactions *via* the production and accumulation of antifungal compounds such as lignin, phenolic compounds, and flavonoids in the infected plants.

The use of nanotechnology in agriculture is increasing at a phenomenal rate, which provides new technologies to enhance crop yields and alleviate the risk of various environmental stresses (Sheteiwy et al., 2015; Hu et al., 2017). Si nanoparticles (SiNPs) display different physio-chemical properties compared with the bulk form. The protection ability of SiNPs against environmental stresses depends on their size, chemical structure, surface covering, and dosage (Alsaeedi et al., 2019). Although many studies have been conducted on the stress-mitigating effect of Si nanoparticles (SiNPs) on plants, little is understood about the role of SiNPs in postharvest handling and vegetable storage. To better test the hypothesis that SiNPs may contribute to postharvest storability of ginger through formation of physical barriers as well as elicitation of defense-related responses and explore the physiology and molecular mechanisms of SiNPs-induced tolerance to postharvest decay, the activity of several defense-related enzymes, the content of bioactive compounds in ginger, including gingerols and flavonoids, the expression levels of genes related to lignin synthesis, the phytopathogen response, and water metabolism were analyzed in this study. The results provide important insights into the mechanisms of SiNP-increased storage life and the potential use of SiNPs as an alternative to synthetic chemicals for the postharvest storage of ginger rhizomes.

## Materials and Methods

### Ginger Handling and Treatment

Ginger rhizomes (*Zingiber officinale* R. cv. Shandongdajiang) were harvested in September (150 d after planting) from Jingzhou, Hubei Province, People’s Republic of China (E:112.026207, N:30.361273). Nanoparticles of silicon dioxide were purchase from Sigma-Aldrich (Lot 637,238). The characteristics of Nano-silicon dioxide were 99.5% purity and 10–20 nm particle size. The rhizomes were delivered to the lab within 2 h. Uniform size rhizomes that had no visible injuries, and vascular discoloration was selected for the analysis. The SiNP (50 mg L^−1^, 100 mg L^−1^, and 150 mg L^−1^) was suspended in water by sonicating the silicon bundles *via* an ultrasonicator at 10 MHz for ∼40 min resulting in a partially homogeneous solution. Approximately 60 kg of rhizomes were evenly divided into four groups and the rhizomes of each group were dipped in distilled water (as the control), SiNPs (SiNP50), 100 mg L^−1^ SiNPs (SiNP100), and 150 mg L^−1^ SiNPs (SiNP150) aqueous solution for 10 min. After being air dried, the rhizomes were uni-packed and stored at 12°C with a relative humidity of 85–90% for 30 d. A total of 20–30 rhizomes from each group were randomly collected at 0, 7, 14, 21, and 28 d after treatment for measurements of the weight loss, the total color difference, firmness, enzyme activities, the total phenolic flavonoid content, lignin content, and qRT-PCR analysis. The rhizome samples were immediately frozen in liquid nitrogen and stored at −80°C until use. For each treatment, there were five biological replications. The rhizomes were collected and used for *in vivo* visualization and a content analysis of the ROS after 30 d of storage.

### Determination of Firmness, Weight Loss, and Total Color Difference

The rhizomes firmness was measured using a TA-XT2i texture analyzer (Stable Micro Systems, Guildford, UK) with a 5 mm cylindrical probe. Each rhizome was tested three times near the central zone. The speed of the probe was 1.0 mm s^−1^ and the penetration depth was 10 mm. The firmness was recorded and expressed as the maximum penetration force (N, Newton). The weight loss was expressed as the percentage loss of the initial weight. The color of the rhizome samples was detected after 10, 20, and 30 d of storage using a Minolta Chroma Meter calibrated with a white standard tile. The results were expressed as the Hunter color values of a^*^, b^*^, and L^*^, a^*^, b^*^, and L^*^ denote lightness, redness and greenness, and yellowness and blueness, respectively. The total color difference among the samples was calculated according to Kha et al. (2010).

### *In vivo* Visualization of the ROS

Histochemical staining of H_2_O_2_ was performed using diaminobenzidine (DAB), and histochemical staining of the superoxide anion radical (O_2_^−^) was performed using nitroblue tetrazolium (NBT), respectively (Zhu et al., 2020).

### Determination of H_2_O_2_, O_2_^−^, and MDA

The MDA content was determined according to the procedures reported by Madhava and Sresty (2000). The OD was recorded at 450, 532, and 600 nm using a spectrophotometer (UV-1800, Shimadzu, Kyoto, Japan). The MDA concentration was determined using a thiobarbituric acid (TCA) reaction as described by Yin et al. (2019). The samples were homogenized in 3 ml of 0.1% (w/v) trichloroacetic acid (TCA) at 4°C. The suspension was centrifuged at 1000 *g* for 20 min, and the supernatant was transferred to a new tube. Then, 2 ml of 0.6% thiobarbituric acid was added to 2 ml of the obtained supernatant. The mixture was thoroughly vortexed and then heated for 20 min in 100°C water bath and cooled immediately in an ice bath. After centrifugation at 7888 *g* for 10 min, the absorbance of the supernatant was read at 450 nm, 532 nm, and 600 nm in the spectrophotometer. The MDA content was expressed as μmol kg^−1^ of the fresh weight.

The H_2_O_2_ contents were assayed according to Gong et al. (2005). The samples were homogenized with 0.1% (w/v) TCA in an ice bath. The extract was centrifuged at 12,000 *g* for 15 min. The reaction mixture (3 ml) contained 0.5 ml of the supernatant, 0.5 ml of 10 mm potassium phosphate buffer (pH 7.0), and 1 ml of 1 M KI, and the absorbance of the reaction mixture was read at 390 nm. The content of H_2_O_2_ was given on a standard curve and was expressed as μmol kg^−1^ of fresh weight. The rate of production of the superoxide anion (O_2_^−^) was determined using a superoxide anion assay kit (Nanjing Jiancheng Bioengineering Institute, China) by measuring the absorbance at 390 nm. The O_2_^−^ generation rate was expressed as nmol h^−1^ kg^−1^ of the fresh weight.

### Determination of Antioxidase Activities

The activity of peroxidase (POD, EC 1.11.1.7), superoxide dismutase (SOD, EC 1.15.1.1), and catalase (CAT, EC 1.11.1.6) were measured according to Shi et al. (2014). Briefly, 1.0 g of fresh samples was homogenized in 6 ml of ice-cold 50 mm sodium phosphate buffer (pH 7.0). The homogenate was centrifuged at 9,661 *g* for 20 min at 4°C. The supernatant was used as an enzyme source to measure POD, SOD, and CAT activities. The protein contents of the enzyme extracts were determined according to the method of Bradford (1976). PPO (EC1.14.18.1) activity was assessed according to Elsherbiny and Taher (2018). The activities of all the enzymes were expressed as 10^6^ U kg^−1^ (unit kg^−1^) of protein.

### Determination of the Total Phenolics and Flavonoid Contents

The total phenolics content and total flavonoid content were extracted and estimated as described by Hatami et al. (2021). Briefly, 2 ml of 7.5% Na_2_CO_3_ solution was added into 0.5 ml of the diluted extract and then mixed with 2 ml of Folin–Ciocalteu’s reagent (10%). The mixture was then incubated at 45°C for 10 min. The absorbance of the samples was measured at 765 nm using a spectrophotometer. Gallic acid monohydrate was used as standard to prepare a calibration curve. The total phenolic contents were expressed as grams gallic acid equivalents per kilogram of the extract on a dry basis.

To determine the total flavonoid contents, 75 μl of 5% NaNO_2_ was incubated with 125 μl of the extraction solution for 6 min. Then, 150 μl of 10% AlCl_3_ was added into the mixture. After reaction for 5 min, 750 μl of 1 M NaOH was added into the solution and placed at room temperature for 15 min. Finally, the absorbance was measured at 510 nm using the spectrophotometer. Quercetin solutions were used to obtain a standard curve. The total flavonoids value was expressed as grams of quercetin equivalents (QE) per kilogram of the extract on a dry basis.

### Determination of the Lignin Content

Lignin was detected quantitatively using a lignin thioglycolic acid method (Qin and Tian, 2005). Approximately, 10 g samples of each treatment were extracted twice in 30 ml of methanol over 24 h; after centrifugation, pellets were collected and dried at 60°C for 24 h, then transferred the obtained residue into sealed screw-cap tubes and added 5 ml of 2 N HCl and 0.5 ml of thioglycolic acid. The mixture was incubated in a water bath for 4 h at 100°C to hydrate the methanol insoluble residue. The extract was centrifuged at 30,000 *g* for 10 min at 20°C and collected pellet was washed once with 5 ml of distilled water. Then, the collected pellet was solubilized in 5 ml of 0.5 N NaOH, sealed with Parafilm, and agitated gently at 20°C for 24 h to extract the lignin thioglycolate. After that, samples were centrifuged at 20°C and 1 ml of concentrated HCl was added to the supernatant solutions. Then, the mixture was stored at 4°C for 4 h to precipitate the lignin thioglycolic acid. The precipitate was collected and used for assessing its absorbance at 280 nm using spectrophotometer. Lignin content was expressed as A280.

### RT-qPCR Analysis

The ginger samples were ground into powder using liquid nitrogen, and the total RNA was extracted using a Plant RNA Extraction Kit (Vazyme, Nanjing, China) according to the manufacturer’s instruction. Then, the qualified RNA was used for cDNA synthesis using the Vazyme PrimeScript RT reagent kit (Vazyme, Nanjing, China). RT-qPCR was conducted on a CFX96 Touch Real-Time PCR Detection System (Bio-Rad, Hercules, CA, United States). Each reaction contained 5 μl of 2× Maxima SYBR Green qPCR Master Mix (Vazyme, Nanjing, China), 2.5 μl of diluted cDNA (400 ng) template, and 10 μM of gene-specific primers; then, nuclease free water was added to 20 μl. The average threshold cycle was obtained to evaluate the relative expression levels using the 2^–ΔΔCt^ method. The primer sequences of the MYB genes were adopted from Li et al. (2020), and other genes’ primer sequences were designed by Primer Premier 5.0 (PREMIER Biosoft International, Palo Alto, CA, United States). The primers sequences used in this study are listed in Supplementary Table 1. Three biological and technical replicates were used for each sample along with a template-free control to check for any contamination.

### *Fusarium solani* Inoculation Assays

In preliminary experiments, the *Fusarium solani* strain was isolated from the surface of naturally decaying ginger rhizomes and identified by the morphology and sequence of the designed specific primers F8 (5′-GCTCAGCGGCTTCCTATTG-3′) and R8 (5′-CGGGGTATTCATCATTCACTTCA-3′). The pathogens strains were cultured on potato dextrose agar (PDA) at 28°C. A spore suspension (1 × 10^8^ spore ml^−1^, counted using a hemocytometer) was prepared by washing 5-d-old sporulating cultures with sterile distilled water. To determine the effect of SiNP100 for controlling the *F*. *solani* infection, the different samples were treated as follows: distilled water (control) and 100 mg L^−1^ of SiNP that contained 0.05% Tween 80. Ten ginger rhizomes were dipped in the different solutions for 10 min. After air drying for 4 h, all ginger rhizomes were wounded with a sterilized borer (1 cm deep × 2 mm wide) at three points around the equator of each fruit. Each wound was pretreated with 50 μl of *F*. *solani* spore suspension (1 × 10^8^ spores ml^−1^). After air drying, all of the treated ginger rhizomes were separately incubated in a plastic box covered with preservative film at 28°C and 90 ± 5% relatively humidity for 8 d.

### Scanning Electron Microscopy

For the Scanning electron microscopy (SEM) analysis, tissue sections of 5 × 5 × 5 mm were fixed and dehydrated according to Ayón-Reyna et al. (2017). The samples were then dried to the critical point by immersing fragments in liquid carbon dioxide in a Samdry-780 Critical Point Dryer (Rockville, Maryland, United States). The dried samples were mounted on aluminum stubs and then sputter-coated with gold at 5 mA and 1.5 kV using a coater (Ion Sputter JFC-1100, Tokio, Japan). The samples were then observed using a JSM-7100F Scanning Electron Microscope (Tokio, Japan).

### Statistical Analysis

Completely randomized design in factorial experiment was used in this study. Data were analyzed using SPSS Statistics (Version19.0, United States). The Student’s *t*-test was used to determine significant differences between mean values at the 0.05 level. There were five replications of each treatment. Data are presented as the means ± standard deviations (SD).

## Results

### SiNP Increased Firmness, Whereas Decreased Water Loss (%) and the Total Color Difference of Fresh Ginger Rhizomes During Postharvest Storage

**Figure 1:**
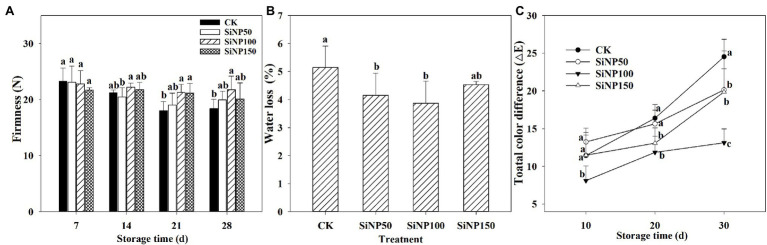
Dynamic change of **(A)** firmness, **(B)** water loss, and **(C)** total color difference in SiNP (50, 100, and 150)-treated ginger rhizomes during postharvest storage. The values are the means of the data from three experiments with five to seven different biological replicates in each experiment ± standard deviations (SD).

The firmness value of ginger rhizomes decreased gradually with time regardless of treatments (Figure 1A). The control ginger rhizomes lost nearly 8.7, 22.6, and 20.9% of their firmness after 14, 21, and 28 days of storage, respectively. Among treatments, the SiNP100-treated ginger rhizomes were the firmest and had lost only 2.5, 6.6, and 4.5% of their firmness after 14, 21, and 28 days of storage, respectively. Additionally, SiNP50 and SiNP100 lost nearly 13.7 and 7.3% of their firmness, respectively, after 28 days of storage.

The maximum water loss (5.15%) after 28 days of the storage was observed in control samples, which were 1.24-, 1.33-, and 1.14-fold higher than the fresh ginger rhizomes treated with SiNP50, SiNP100, and SiNP150, respectively. SiNP100 exhibited the minimum water loss (3.87%) at the end of storage (Figure 1B).

The total color differences of different treatments are shown in Figure 1C. Compared with control, the SiNP100 treatments decreased the total color difference throughout the entire storage period. Additionally, SiNP50 decreased the total color difference after 30 days’ storage, and SiNP150 largely decreased the total color difference after 20 and 30 days’ storage.

### SiNP Decreased the Rhizome Decay and Alleviated the ROS Burst

**Figure 2:**
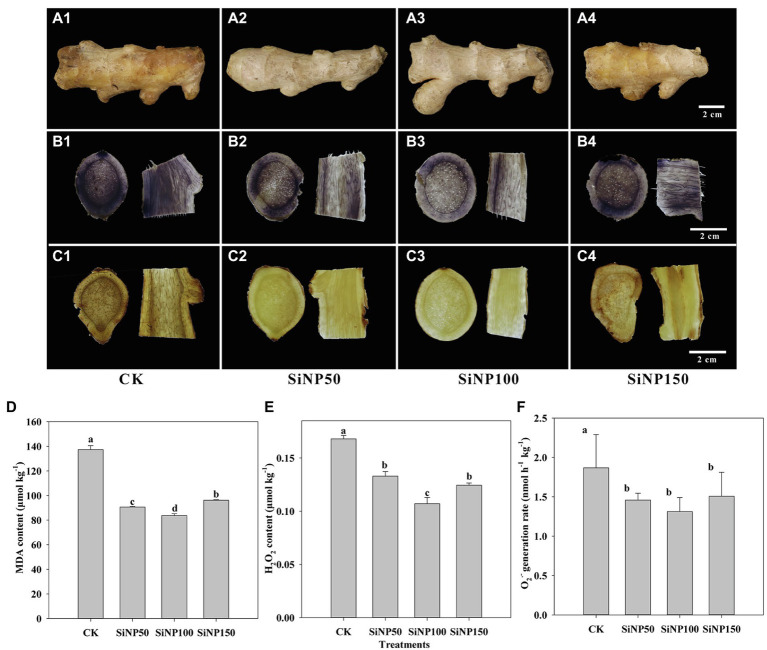
Dynamic change of **(A)** external features, and the *in vivo* visualization of **(B)** O_2_^−^ and **(C)** H_2_O_2_ and the contents of **(D)** MDA, **(E)** H_2_O_2_, and **(F)** O_2_^−^ in SiNP (50, 100, and 150)-treated ginger rhizomes during postharvest storage. The values are the means of the data from three experiments with five to seven different biological replicates in each experiment ± standard deviations (SD).

Compared with the control, SiNP treatment delayed the decay of fresh rhizomes after 28 days’ storage, with the SiNP100 being the best, followed by SiNP50 and SiNP150 (Figure 2A). For visualization of the O_2_^−^ and H_2_O_2_, histochemical analyses were performed on day 28 of storage. Intensive dark spot staining of the DAB reagent, characteristic of blue formazan precipitates, was observed in the control samples. Rhizomes treated with all SiNP concentrations showed weaker dye staining compared with the control, with SiNP100 being the weakest, followed by SiNP50 and SiNP150 (Figure 2B). The intensity of NBT staining, the appearance of brown spots characteristic of the reaction of DAB with H_2_O_2_, was observed in the control samples. Similarly, rhizomes treated with SiNP100 showed the weakest dye staining compared with control. This was followed by SiNP50 and SiNP100 (Figure 2C).

The MDA content was decreased when treated with different concentrations of SiNP (Figure 2D). As shown in Figures 2E,F, SiNP treatments greatly reduced the H_2_O_2_ content and O_2_^−^ generation rate after 30 days of storage compared with control, with SiNP100 being the lowest, followed by SiNP50 and SiNP150.

### SiNP100 Effects on the Activity of SOD, CAT, POD, and PPO

SiNP100 (100 mg L^−1^ of SiNP) showed the best effect in extending the storage life of ginger; hence, this dose was adopted to conduct the following experiments.

**Figure 3:**
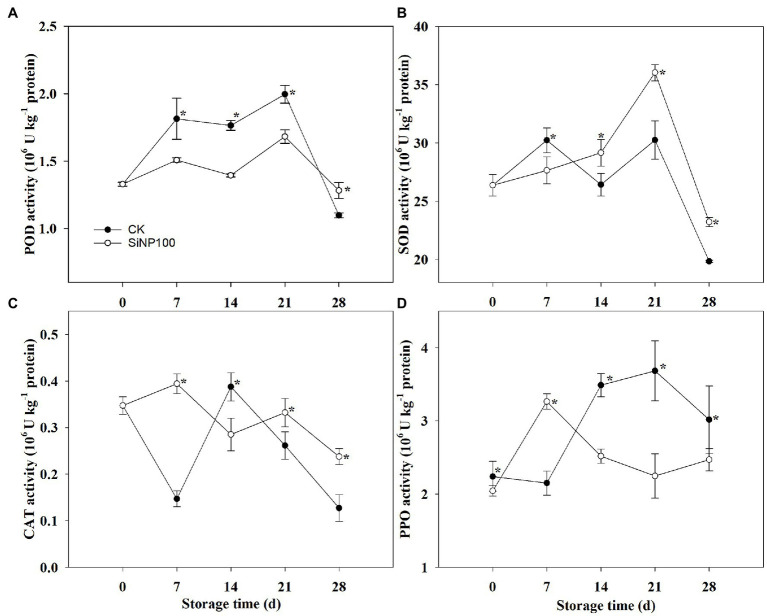
Dynamic change of **(A)** POD, **(B)** SOD, **(C)** CAT, and **(D)** PPO activities of SiNP100-treated ginger rhizomes during postharvest storage. The values are the means of the data from three experiments with five to seven different biological replicates in each experiment ± standard deviations (SD).

POD activity in the control ginger rhizomes increased at 0–21 d but decreased at 28 d, and two peaks were determined at 7 and 21 d. The POD activity in both the SiNP100-treated and the control ginger rhizomes peaked at 7 and 21 d (Figure 3A). The POD activity of the ginger rhizomes treated with SiNP100 was lower than of the control at 0–21 d of the storage period, but higher than that of the control on the day 28 (Figure 3A).

SOD activity increased at 7 and 21 d but decreased at 14 and 28 d in the control. The SiNP100 treatment largely increased the SOD activity after 14 d of treatment compared with control. In the SiNP100-treated ginger rhizomes, the highest level of SOD activity was determined at 21 d and that was 1.2 times higher than that of the control (Figure 3B).

In the control samples, the activity of CAT declined sharply on 7 d and then increased on 14 d, followed by a sharp decrease at 21 and 28 d. The maximum CAT activity (0.39) was observed at 14 d; while the minimum (0.13) was recorded at the end of 28 days’ storage. The CAT activities of the ginger rhizomes treated with Si100 showed a decreasing trend during the storage period. Ginger rhizomes treated with SiNP100 had higher CAT activities at 7, 21, and 28 d compared with control (Figure 3C).

As shown in Figure 3D, the activity of PPO in the control ginger rhizomes increased gradually during the storage period and peaked at 21 d. PPO activity in the SiNP100-treated ginger rhizomes peaked at 7 d (higher than the control) and then decreased in the following assay period (lower than the control; Figure 3D).

### SiNP100 Effects on the Total Flavonoid Content and the Total Phenol Content

**Figure 4:**
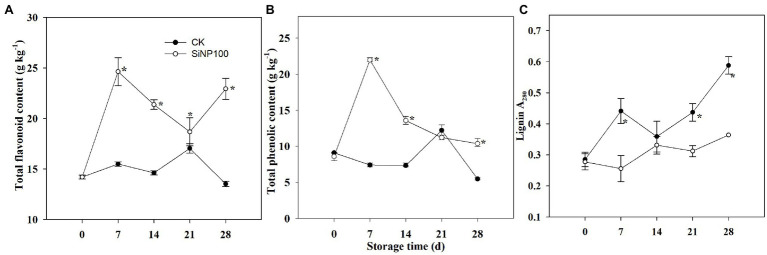
Dynamic change of **(A)** total flavonoid, **(B)** total phenolic, and **(C)** lignin content of SiNP100-treated ginger rhizomes during postharvest storage. The values are the means of the data from three experiments with five to seven different biological replicates in each experiment ± standard deviations (SD).

As shown in Figure 4A, the SiNP100 treatment increased the total flavonoid content throughout the experiment compared with the control ginger rhizomes. SiNP100 increased the total flavonoid content by 59.0, 46.3, 9.8, and 69.6% compared with that of the control.

The total phenol content was notably enhanced by the SiNP100 treatment at 7, 14, and 28 d (Figure 4B). The total phenol content showed a sharp increase before 7 d of storage and then declined, and the total phenol content activities in the Si-treated ginger rhizomes were 197.2, 83.3, and 90.8% higher than those in the control at 7, 14, and 28 d, respectively.

As shown in Figure 4C, the SiNP100 treatment increased the lignin content throughout the experiment compared with the control ginger rhizomes. SiNP100 increased the total flavonoid content by 59.0, 46.3, 9.8, and 69.6% compared with that of the control.

### SiNP100 Effects on the Expression of MYB, LysM, and AQP Genes

**Figure 5:**
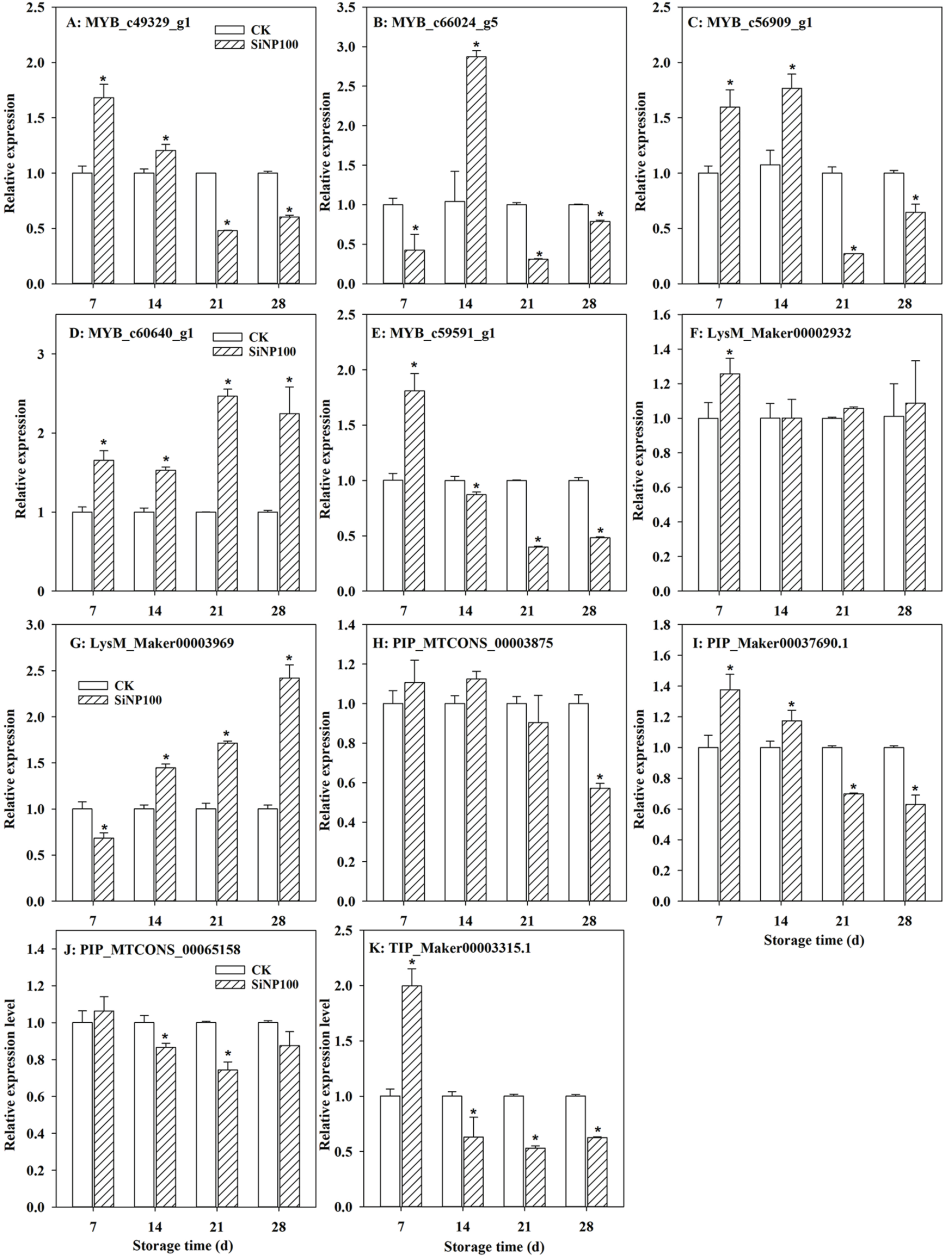
Alternation of *MYB* **(A–E)**, *LysM* **(F–G)**, and *AQPs* **(H–K)** transcripts in SiNP100-treated ginger rhizomes during postharvest storage. The error bars represent the standard deviation of the mean of three replicates. The values are the means of the data from three experiments with five to seven different biological replicates in each experiment ± standard deviations (SD).

Lignin biosynthetic genes has been proved to be regulated by MYB transcription factors (Li et al., 2020). In this study, five MYB transcription factors were selected to perform RT-qPCR. The treatment with Si100 resulted in inducible effects on the expression of *MYB_c49329_g1* and *MYB_c56909_g1* at 7 and 14 d compared with the control (Figures 5A,C). After 21 d, the expression levels of *MYB_c49329_g1* and *MYB_c56909_g1* decreased. The SiNP100 treatment caused an increase in the expression of *MYB_c66024_g5* and *MYB_c59591_g1* at 7 and 14 d, respectively, but decreased their expression levels at other time points (Figures 5B,E). The expression level of *MYB_c60640_g1* increased due to the Si100 treatment throughout the experiment (Figure 5D).

LysM (lysine motif) receptor kinases have been reported to play an important role in chitin signaling and fungal resistance (Brulé et al., 2019). The expression levels of two ginger LysM genes were analyzed. Compared with the control, SiNP100 largely increased the expression level of *LysM_Maker00002932* at 7 d (Figure 5F). The expression levels of *LysM_Maker00003969* increased along with the treatment duration. Its expression level decreased 0.68-fold at 7 d but increased 1.45-, 1.71-, and 2.42-fold at 14, 21, and 28 d, respectively (Figure 5G).

Aquaporins (AQPs) mediate water balance in plant cells and have multiple roles in the fruit ripening and postharvest storage processes (Alleva et al., 2010; Zhang et al., 2021b). The transcript levels of all four AQPs genes in the ginger rhizomes showed similar expression patterns with SiNP100 treatment, which were firstly enhanced and then decreased compared with the control. Compared with the control, the expression level of *PIP_MTCONS_00003875* was slightly increased at 7 and 14 d with SiNP100 treatment but then decreased after 21 d (Figure 5H). SiNP100 increased the expression level of *PIP_Maker00037690.1* after 7 and 14 d of treatment but decreased it after 21 d of treatment (Figure 5I). SiNP100 increased the expression levels of *PIP_MTCONS_00065158* and *TIP_Maker00003315.1* at 7 d and then decreased their expression levels after 14 d of treatment (Figures 5J,K).

### SiNP100 Effects on the Infection of *F. solani*

**Figure 6:**
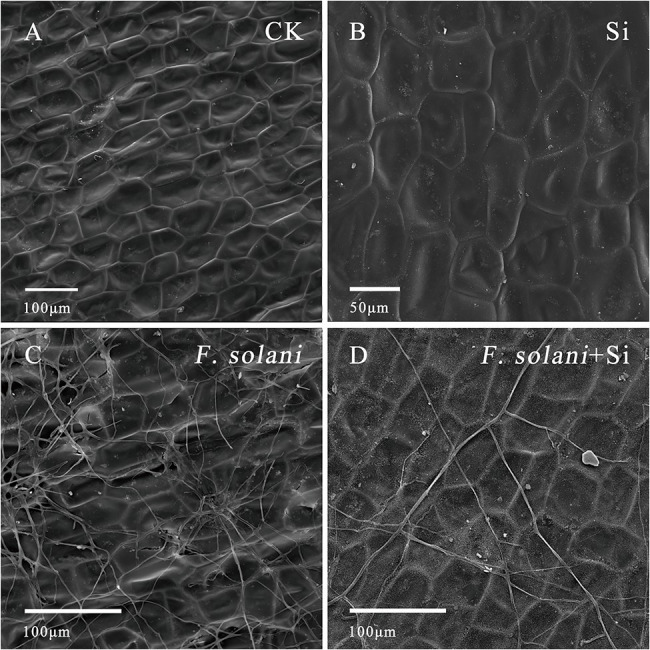
Scanning electron microscopy photomicrographs of the **(A)** control, **(B)** treated with SiNP100, **(C)** *F. solani* infection, and **(D)** *F*. *solani* infection and treated with SiNP100.

Scanning electron microscopy (SEM) observations of *F*. *solani* colonization were conducted 3 d after inoculation. Epidermal cells are neatly arranged in CK samples (Figure 6A), while obvious white Si depositions were observed in the Si and *F*. *solani* + Si treatments (Figure 6B). Hyphae could be seen growing superficially along the sample surface (Figure 6C) and SiNP100 treatment largely decreased the growth of hyphae and prevented the hyphae from penetrating into cells through the formation of white Si layers (Figure 6D).

## Discussion

The postharvest decay and senescence of fruit and vegetables cause a large amount of economic losses worldwide. Si has been reported to be effective against postharvest decay and diseases inducing resistance and fungistatic effects are a promising alternative tools to chemical fungicides (Elsherbiny and Taher, 2018; Lyu et al., 2019). Fruit and vegetables generally show a softening process during postharvest storage, which is a primary cause of quality loss. The results of this study showed that Si was effective to maintain firmness, decrease water loss, and decrease the total color difference of ginger rhizomes, with the regulatory effect of SiNP100 being the best (Figure 1). Based on these results and the results of previous studies in other plant species, such as rice (Gong et al., 2005), cucumber (Zhu et al., 2015; Yin et al., 2019), and muskmelon (Lyu et al., 2019), the mechanisms for Si enhanced firmness and water content may be due to the following (1) A physical barrier forms due to the Si deposition (Ma and Yamaji, 2008) and improves the mechanical properties due to the hemicellulose-bound form of Si (He et al., 2015); (2) lower transpiration and/or water loss rates may be regulated by AQPs (Zhu and Gong, 2014; Zhu et al., 2015). As these activities impart to an increase in firmness and reduce moisture loss from fresh ginger, they ultimately in turn control color change during storage. Overall, the results related to quality attributes indicate that SiNPs is a promising tool to increase and maintain fruit quality properties during postharvest storage.

ROS could serve as a signal molecule in postharvest fruits and vegetables for activating defense mechanisms during plant/pathogen interactions. However, excess ROS could cause oxidative damage, resulting in metabolic disturbance (Li et al., 2012; Zhu et al., 2020). A close relationship between the ROS content and fruit senescence has been reported (Chu et al., 2018). In this study, H_2_O_2_, O_2_^−^, and the MDA contents in ginger rhizomes slices were decreased by using silicon treatment, especially SiNP100, indicating that Si treatment could enhance the capacity of ginger rhizomes to resist senescence-induced oxidative stress, and thereby delay the postharvest senescence of rhizomes (Figure 2). However, in harvested muskmelon, sodium silicate treatment reduced the decay caused by *Trichothecium roseum* through increasing the content of ROS (OH, H_2_O_2_, and O_2_^−^) and stimulating the antioxidant system (Li et al., 2012; Lyu et al., 2019). Hence, these seemingly contradictory findings could be due to the dual function of ROS, which could function as a signal molecule in the environmental stress response, while excess ROS production could seriously disrupt normal metabolism (Marschall and Tudzynski, 2016). In addition, different Si sources and hosts, such as sodium silicate and nano-Si, were used in these studies, which may have different regulation mechanisms (Zhu et al., 2019a). Finally, the combined effect of Si treatment and pathogen challenge reported in harvested muskmelon (Li et al., 2012; Lyu et al., 2019) may be distinct from the present study, the decay of which may have been caused by latent infections established in the field or wound infections during subsequent harvesting and handling.

Plants have developed an antioxidant defense system that includes antioxidant enzymes (e.g., SOD, POD, and CAT) and nonenzymatic components (e.g., phenolic compounds, ascorbic acid, and carotenoids) to deal with the undesired accumulation of ROS. SOD was a main source of H_2_O_2_ *via* dismasting superoxide radical, whereas CAT, subsequently convert H_2_O_2_ to H_2_O (Wang et al., 2019). Li et al. (2020) reported that postharvest dehydration caused oxidative damage and resulted in the accumulation of ROS and MDA, and affect postharvest quality of ginger rhizomes. Similarly, the present study indicated that ROS and MDA accumulated after long-term storage. SiNP100 treatment activated SOD and CAT activity in ginger rhizomes to maintain the ROS balance especially after 14 d of treatment. During the later storage period, lower levels of O_2_^−^, H_2_O_2_, and MDA in the samples with Si100 treatment suggested better protection from oxidative damage, which might be attributed to the higher SOD and CAT activities.

PPO catalyzes oxidation of polyphenols into quinones, which are highly toxic to many species of fungi and can induce lignification in plant cells during the microbial infection (Zhang et al., 2018; Lyu et al., 2019). The accumulation of POD might participate in cell wall synthesizing processes, oxidation of phenolic compounds to quinones in the presence of H_2_O_2_, and the lignification process of plant cells during plant-fungal pathogen reactions (Elsherbiny and Taher, 2018). Increased POD and PPO activities have been reported to contribute to the enhancement of disease resistance in postharvest vegetables and fruits (Elsherbiny and Taher, 2018). Silicon addition enhanced the activities of POD, PPO, and phenylalanine ammonia-lyase (PAL) in carrot roots inoculated with *S*. *sclerotiorum*, and thus promoted the defense response in carrots during storage (Elsherbiny and Taher, 2018). However, in this study, the activities of POD and PPO were generally lower in the SiNP100 treated samples compared with control (Figure 3). To be specific, POD activity was lower in the SiNP100-treated samples throughout the experiment, except for a slight incense at 28 d. PPO activity was stimulated by SiNP100 after 7 d of treatment and then decreased from 14 d onward. We speculated that the Si treatment may delay latent infections established in the field or wound infections during subsequent harvesting and handling; thus, increases in certain types of antioxidant enzymes were not required to prevent damage.

Phenolics and flavonoids are important natural bioactive compounds in plants. They are also a type of antioxidant substance capable of scavenging free superoxide radicals (Haider et al., 2020). It was found that the pharmacological activities of ginger were primarily attributed to phenolic and flavonoid components. Studies have suggested that higher accumulations of total phenolics and flavonoid were correlated with higher antioxidant capacity, while oxidation of phenolic compounds is the primary cause of browning in vegetables and fruit (Liu et al., 2018; Haider et al., 2020). In this study, the total phenolics and flavonoid content decreased after 28 d of treatment compared with SiNP100 treatment, which have been partially attributed to the increased total color difference, as shown in Figure 4. Moreover, a decreased total phenolics and flavonoid content are not good for the taste and pharmacological activities of ginger rhizomes. The Si addition enhanced the total phenolic and flavonoid content of ginger at both the early stage (7 d) and the later stage (28 d) of storage, implying higher antioxidative ability (Figure 4). The higher total phenolic and flavonoid content could be attributed to lower activities of POD and PPO in the SiNP100-treated samples compared with the control. This conclusion agrees with that of previous studies in mushroom (*Agaricus bisporus*) and lemon fruit (*Citrus limon*) where it was found that salicylic acid (SA) may increase the synthesis of total phenolics by inhibiting the activity of POD and PPO (Siboza et al., 2014; Dokhanieh and Aghdam, 2016). Taken together, at the later period of storage, the decrease in ROS contents in the SiNP100 treatment samples could have been related to an increase in the activities of antioxidant enzymes (e.g., CAT and SOD) and in the contents of nonenzymatic antioxidants contents (total phenolics and flavonoid). This would also alleviate membrane lipid peroxidation (MDA), as well as color changes, in ginger rhizomes. The results also showed good potential for the use of Si for increasing phenolic compounds in ginger rhizomes with high antioxidant activities and better rhizomes quality.

Lignin is the terminal product in the phenylpropanoid pathway that synthesizes diversified phenolic compounds and is a major component of the vascular plant cell wall (Zhang et al., 2021a). Lignin provides plants with rigidity that protects plants from pathogenic infections. Nevertheless, deposition of lignin negatively influences the postharvest quality of crops (Jin et al., 2021). Previously, Li et al. (2020) found that lignin accumulated in ginger rhizomes after dehydration stress. In carrots, lignin accumulated under dehydration and wounding stress works to prevent water loss (Becerra-Moreno et al., 2015). In this study, the lignin content in the Si-treated ginger did not increase compared with the control. This was in consistent with the activity of POD, which is crucial for lignin synthesis. Jin et al. (2021) reported that 20 mg L^−1^ of abscisic acid treatment slightly stimulated lignin accumulation in the peel of kiwifruit and prevented lignin accumulation in the pulp. Thus, whether exogenous Si treatment exerted effects for controlling lignification in different sections of ginger rhizomes needs to be further explored. MYB transcription factors (TFs) are involved in many developmental and physiological processes and have been reported to regulate lignin biosynthesis (Chen et al., 2021). In the present study, the expression levels of most of the selected MYB were induced by SiNP100 at 7 and/or 14 d but were decreased by SiNP100 at 21 and 28 d. These results suggest that MYB transcription factors may participate in Si-regulated physiological changes during postharvest storage. The expression levels of the selected MYB were induced by Si100 at 7 and/or 14 d, which seems to be inconsistent with the content of lignin. Further studies are required to explore the exact function of these MYB genes in regulating lignin biosynthesis, e.g., activator or repressor, as well as in other regulatory networks that regulate physiological and biochemical processes.

During postharvest storage, water loss largely affects vegetable and fruit quality, including size and firmness as well as other physiology metabolisms such as enzymatic enzyme activities. AQPs play a vital role in the transport of water across vacuolar and plasma membranes (Zhu et al., 2019b). Silicon has been reported to alleviate water stress by regulating the expression of AQPs. As SiNP100 greatly decreased the water loss of ginger rhizomes during postharvest storage, we analyzed the effect of SiNPs on four selected AQPs expression profiles of ginger. SiNP100 addition induced the expression levels of all selected AQPs (3 PIPs and 1 TIP) at 7 and/or 14 d of treatment but greatly decreased their expression levels after 21 d of treatment (Figure 5). Zhang et al. (2021b) proposed that a downregulated expression of *AQPs* (particular PIPs and TIPs) in sweet orange could reduce hydraulic conductance, therefore minimizing water movement in response to water loss after postharvest. Hence, the alteration in *AQPs* expressions with Si addition in this study may be an effective strategy in ginger rhizomes to deal with water loss and maintain water status during storage. The results suggested that Si-mediated water loss is not simply due to Si deposition on the surface of ginger rhizomes but is performed through more complex physiology and molecular mechanisms that required further study.

In addition to water loss, microorganisms are important causal agents of spoilage in postharvest fruits and vegetables (Bi et al., 2007; Palou, 2018; Wang et al., 2020). In this study, several fungi were isolated from the decayed ginger rhizomes, among which *F*. *solani* was the main primary fungi that causes latent infections. The inhibitory potential of Si against *F*. *solani* was investigated under *in vivo* conditions. These results suggested that Si seemed to prevent hyphae penetration into cells through the formation of white Si layers on the cell surface. Elsherbiny and Taher (2018) proposed that Si could decrease *S*. *sclerotiorum* caused by postharvest carrot rot by exerting an effect on the pathogen itself (e.g., growth, sclerotia, cell membrane permeability, lipid peroxidation, and oxalic acid content), as well as indirectly promoting the defense response. Whether this is the case in the effect of Si on *F*. *solani* affected ginger rhizomes requires further study, as we did not observe reduced spore germination with Si addition under *in vitro* and *in vivo* conditions. Overall, the results of this study showed that in postharvest ginger rhizomes, SiNP may be effective to (1) maintain firmness of by decreasing water loss *via* modulating AQPs expressions; (2) increase better rhizomes quality by increases in certain types of antioxidant enzymes and natural bioactive compounds; and (3) regulate transcript levels of genes related to phenylpropanoid pathway like MYB and defense-related responses.

## Conclusion

In the present study, our results revealed that SiNP100 treatment was most effective in improving qualities of ginger rhizomes during postharvest storage. SiNP100 treatment increased the firmness of ginger rhizomes, decreased weight loss, and decreased the total color difference. Furthermore, SiNP100 treatment promoted the SOD and CAT activities and increased the accumulation of total flavonoids and phenolics, resulting in higher antioxidant capacity. POD and PPO activities were decreased by SiNP100 treatment, which may have contributed to the decreased total color difference and lignin content. Moreover, the SiNP100 treatment participated in the expression regulation of several MYB, LysM, and AQP genes, suggesting its possible roles in lignin synthesis, phytopathogen responses, and water metabolism. These results confirm that SiNPs can be applied as an alternative tool to chemical fungicides for the maintenance of ginger quality and to control postharvest diseases in ginger.

## Data Availability Statement

The datasets presented in this study can be found in online repositories. The names of the repository/repositories and accession number(s) can be found in the article/Supplementary Material.

## Author Contributions

YZ, YL, and JY conceived and designed the experiment. HP and KX performed all treatments and collected the samples. HP, KX, and XZ performed the physiological indexes analysis. JZ performed the SEM analysis. HP performed the RT-PCR analysis. YZ and HH wrote the manuscript. FG contributed to improving and revising the final version. All authors contributed to the article and approved the submitted version.

## Funding

This research was funded by the Natural Science Foundation of Hubei Province (2021CBF512), Scientific Research Program of Hubei Provincial Department of Education (no. D20201301), and Key Research and Development program of Hubei province (2021BBA096).

## Conflict of Interest

The authors declare that the research was conducted in the absence of any commercial or financial relationships that could be construed as a potential conflict of interest.

## Publisher’s Note

All claims expressed in this article are solely those of the authors and do not necessarily represent those of their affiliated organizations, or those of the publisher, the editors and the reviewers. Any product that may be evaluated in this article, or claim that may be made by its manufacturer, is not guaranteed or endorsed by the publisher.
