# Supplementary material for: Silicon Nanoparticles Enhance Ginger Rhizomes Tolerance to Postharvest Deterioration and Resistance to Fusarium solani
Source: Front Plant Sci. 2022 Mar 15;13:816143. doi: 10.3389/fpls.2022.816143 (PMC8965286; doi:10.3389/fpls.2022.816143)
Supplement: Supplementary file 1 [file Table_1.DOCX]

Supplementary Material

# Supplementary Figures and Tables

## Supplementary Figures

**Supplementary Table 1.** Detailed information of primers used for RT-qPCR.

| Gene | Accession number. | Primer sequence(5' to 3') | Product size (bp) | Tm | GC% |
| --- | --- | --- | --- | --- | --- |
| MYB-c49329_g1 | c49329_g1 | F：AGCGTGGATGACATCTGGTC | 157 | 57.45 | 55 |
|  |  | R：TGGGAGGAAGCCACAGTGAT |  | 58.84 | 55 |
| MYB-c66024_g5 | c66024_g5 | F：ATTTGATGCATGCGCCATCG | 194 | 56.95 | 50 |
|  |  | R：GCTCTCCATCTCGTCTTCCG |  | 58.05 | 60 |
| MYB-c56909_g1 | c56909_g1 | F：TCGGCATGGAGTTGGATCAC | 147 | 57.75 | 55 |
|  |  | R：GTTGCGCGGATGTATGTGTG |  | 57.39 | 55 |
| MYB-c60640_g1 | c60640_g1 | F：ATCCCATGGACCGAAGAGGA | 138 | 58.11 | 55 |
|  |  | R：ATACTTTTGCGCGTGGCTTG |  | 56.72 | 50 |
| MYB-c59591_g1 | c59591_g1 | F：GATCAGCCGGTCTATTCCCG | 196 | 58.24 | 60 |
|  |  | R：CTTTATGGCGTTGTCGGTGC |  | 57.39 | 55 |
| LysM-Maker00002932 | Maker00002932 | F：CAACAAAATTGGCCGGGGTG | 197 | 60.6 | 55 |
|  |  | R：GATTGGCGCCTTCAACACAG |  | 60.11 | 55 |
| LysM-Maker00003969 | Maker00003969 | F：ATTTGGGGCGAAAACTCCCA | 195 | 60.18 | 50 |
|  |  | R：TGGCACACGGAGTTGCTATT |  | 59.96 | 50 |
| PIP-MTCONS_00003875 | MTCONS_00003875 | F：AGCCAGGACAAGGACTACAAG | 75 | 56.75 | 52.38 |
|  |  | R：GAAGGACCAGGAGGTGAGTT |  | 56.63 | 55 |
| PIP-Maker00037690.1 | Maker00037690 | F：GAAGGAGGAGGATGTGAAGC | 146 | 55.47 | 55 |
|  |  | R：CCCTGTAGAAGGACCACGACT |  | 58.83 | 57.14 |
| PIP-MTCONS_00065158 | MTCONS_00065158 | F：AAGGTGTCGCTGGTGAGG | 109 | 58.05 | 61.11 |
|  |  | R：CAAAGGTGTTGTACTGGTGCT |  | 55.3 | 47.62 |
| TIP-Maker00003315.1 | Maker00003315 | F：ATGGCCGCTGAGAAGATGATG | 179 | 57.68 | 52.38 |
|  |  | R：GAACGCACGACGCTGATGT |  | 58.94 | 57.89 |
